# Hepatitis B virus and hepatitis C virus in pregnant Sudanese women

**DOI:** 10.1186/1743-422X-4-104

**Published:** 2007-10-24

**Authors:** Rasha M Elsheikh, Ahmed A Daak, Mohamed A Elsheikh, Mubarak S Karsany, Ishag Adam

**Affiliations:** 1 Department of Obstetrics & Gynecology, Faculty of Medicine, University of Khartoum, Khartoum, Sudan; 2 Department of Pathology, Faculty of Medicine, Juba University, Khartoum, Sudan

## Abstract

**Background:**

The epidemiology of viral hepatitis during pregnancy is essential for health planners and programme managers. While much data exist concerning viral hepatitis during pregnancy in many African countries, no proper published data are available in Sudan.

**Aim:**

The study aimed to investigate the sero-prevalance and the possible risk factors for hepatitis B virus (HBV) and hepatitis C virus (HCV) among antenatal care attendants in central Sudan.

**Methods:**

During 3 months from March–June 2006, sera were collected from pregnant women at Umdurman Maternity Hospital in Sudan, and they were tested for markers of hepatitis B virus (HBVsAg) and HCV.

**Results:**

HBVsAg was detected in 41 (5.6%) out 728 women, Anti-HCV was detected in 3 (0.6%) out of 423 women, all of them were not aware of their condition. Age, parity, gestational age, residence, history of blood transfusion, dental manipulations, tattooing and circumcision did not contribute significantly to increased HBVsAg sero-positivity.

**Conclusion:**

Thus 5.6% of pregnant women were positive for HBVsAg irrespective of their age, parity and socio-demographic characteristics. There was low prevalence of Anti-HCV.

## Introduction

Hepatitis B virus (HBV) infection affects over 350 million people worldwide and over one million die annually of HBV-related chronic liver disease. In endemic areas, most individuals are infected by vertical transmission, or in the early childhood [1]. Hepatitis C virus (HCV) infection is a major worldwide public health problem. The World Health Organization (WHO) estimates that 3% of the world's populations are chronically infected with HCV, most of these cases occur in Africa, which is reported to have the highest HCV prevalence rate [2,3]. Although, direct percutaneous inoculation is the most efficient mode of transmission of HCV, several studies have demonstrated that sexual, household, occupational, and vertical transmission of HCV may also be of importance [4].

Viral hepatitis during pregnancy is associated with high risk of maternal complications, has a high rate of vertical transmission causing fetal and neonatal hepatitis and it has been reported as a leading cause of maternal mortality in Sudan [5-8]. The basic epidemiological data for these viruses might be of great importance to the programme mangers and health planners, so as to initiate the relevant vaccine and screening packages in the antenatal care clinics. While, much data exist about the epidemiology of viral hepatitis during pregnancy in other African countries [9-13], no proper published data are available in Sudan, which is the largest country in Africa. Thus, the current study aimed to investigate the prevalence and the possible risk factors for HBV and HCV among antenatal attendant in central Sudan.

## Methods

This was across-section study conducted at Umdurman maternity hospital, Sudan during the period of March–June 2006. After an informed consent all pregnant women attended the first antenatal care visit were approached to participate in the study. A fixed questionnaire was applied to gather relevant socio-demographic characteristics of these women (age, education, gestational age, parity, residence, occupation). Then the possible risk factors (history of surgery or blood transfusion, tattooing, circumcision etc) for HBV were inquired for. After immediate centrifugation, the sera were tested for HBVsAg anti-HCV using ELISA.

### Ethics

The study was approved by the Ethics committee of the Faculty of Medicine, Khartoum University and an informed consent was taken from the women.

### Statistics

The data were entered in computer and double checked before analysis by SPSS for windows. The means, percentages were calculated and compared between the sero-positive and sero-negative (HBVsAg) using student t-test and chi-square test respectively. Multiple regression was used, sero-positive for HBVsAg as dependent variable and the age, parity, education and the other possible risk factors as independent variables. *P *> 0.05 was considered significant.

## Results

### General characteristics of the women

During the study period 728 women were enrolled at 32.1 weeks of gestational age. Their mean (SD) age was 27. 3 (6.2) years. The mean (SD) of the parity was 2.2(1.6), 128 (17.5%) of them were primigravidae. More than one third, 289(40%) of these women had less than secondary level education. 141(19.3%), 63 (8.8%) women gave history of jaundice and blood transfusion, respectively. 69(9%) and 21(3%) had traditional scares and tattooing, respectively. One third of these women (231) had history of dental maneuvers.

HBsAg was detected in 41 (5.6%) out of 728 women, all of them were not aware of their condition. The mean (SD) of the age, parity and gestational age were not significantly different between the sero-positive and sero-negative women (data not shown).

Table 1, showing the results of the univarite and multivariate analysis. None of the expected risk factors (parity, age, history of blood transfusion, dental manipulations, tattooing and circumcision) had been found to be associated with HBVsAg sero-positivity. Due to kids constrains, anit-HCV was tested in the first 423 women. Three (0.6%) out of these were found to be positive.

**Table 1:** Showing the univarite and multivarite analysis for the possible risk factors for HBV sAg among pregnant women.

Variables	Univarite analysis			Multivarite analysis		
	O R	95% CI	P	O R	95% CI	P

Primigravidae	1.3	0.5–3.1	0.5	1.0	0.4–2.9	0.8
Age ≤27 years	0.9	0.48–1.7	0.7	0.89	0.3–1.9	0.7
Education < secondary school	0.5	0.3–1.0	0.07	0.6	0.2–1.5	0.3
Blood transfusion	1.8	0.75–4.6	0.1	1.9	0.7–5.6	0.19
Surgical operation	1.0	0.3–2.6	0.9	1.5	0.49–4.5	0.4
Circumcision	0.55	0.23–1.2	0.1	0.66	0.22–1.9	0.4
Home delivery	0.9	0.44–1.9	0.8	0.67	0.26–1.7	0.4
Dental manipulation	0.3	0.2–1.2	0.3	0.3	0.12–1.2	0.2
History of jaundice	1.7	0.8–3.5	0.1	1.7	0.7–4.2	0.22

## Discussion

Perhaps, this is the first published study documenting sero-prevalence of HBV and HCV among pregnant Sudanese women. Around 5% and less than one percent of these women had been found to be positive for HBVsAg and HCV respectively. Interestingly, this prevalence is much lower in comparison with the prevalence in other African countries [9,11-14]. However, comparison between our study and the others' should be taken cautiously. Firstly different methods had been applied, in our study we aimed to detect antibodies using ELSIA, while some of these studies, DNA of these viruses had been detected rather than antibodies. Secondly the differences in socio-demographic background of these women have to be remembered. Thirdly, the difference in prevalence and interactions of HBV and HCV and HIV might explain the results. Yet, we have recently, documented HIV prevalence in 1% of Sudanese pregnant women of central Sudan [15]. Furthermore, co-infection of HIV and HBV/HCV seems to demonstrate a correlation between these two infections, which could influence the evolution of these diseases [11,12]. Parallel and overlapping HIV and blood borne hepatitis epidemics in Africa and Influence of maternal (HIV) co-infection on vertical transmission of HCV have been reported before [16,17]. Thus, the geographical influence of high endemicity in neighboring sub-Saharan countries might change the current situation in the future. Furthermore, even inside Sudan higher prevalence of HBV and HCV had been reported among non-pregnant population of the southern and central Sudan [18,19].

Unlike the previous reports [14,20], none of the expected risk factors (age, parity and the other socio-demographic characteristics) for sero-positivity for HBVsAg had been identified in the current study. The explanations for such need to be explore in the future. Other studies are urgently needed to investigate HCV and HIV co-infections and their vertical transmission. Other viruses like hepatitis E should be investigated among the whole population as well as in pregnant Sudanese women.

## Conclusion

Thus, 5.6% of pregnant women were positive for HBVsAg irrespective to their age, parity and socio-demographic characteristics. There was a low prevalence of Anti-HCV.

## Authors' contributions

RME and AAD carried out the clinical study and participated in the statistical analysis and procedures, MAE participated in the analysis, IA coordinated and participated in the design of the study, statistical analysis and the drafting of the manuscript. MSK and AAD participated in the lab work. All the authors read and approved the final version.
